# Effects of Oil of Tansy

**Published:** 1881-12

**Authors:** 


### Effects of Oil of Tansy.

Dr. G. Jewett, Boston *Medical and Surgical Journal*, reports eight cases of poisoning with this drug. Case 1—Fifteen drops at 11 A.M., a teaspoonful at 2 P.M.; convulsions, shock, general cyanosis; recovery. Case 2—Teaspoonful to promote catamenia; convulsions and death in one hour and a half. Case 3—Unknown quantity to cause abortion; convulsions; death in three hours and a quarter; no abortion. Case 4—Teaspoonful to cause abortion; coma, recovery; no abortion. Case 5—Four drachms; spasms and death. Case 6—To cause abortion; rapid death; no abortion. Case 7—Decoction of tansy leaves to produce abortion; paralysis, coma, death in twenty-four hours without abortion. Case 8—Infusion of leaves daily for a week; also for vaginal injection; abortion, metritis, peritonitis; recovery after three months. As druggists are often asked for oil of tansy under various pretenses, we believe the above table will be useful in reminding them of the dangers attending the sale of tansy and its preparations.—*Country Practitioner.*—*Therapeutic Gazette.*

